# A Brain‐To‐Brain Mechanism for Social Transmission of Threat Learning

**DOI:** 10.1002/advs.202304037

**Published:** 2023-08-06

**Authors:** Yafeng Pan, Mikkel C. Vinding, Lei Zhang, Daniel Lundqvist, Andreas Olsson

**Affiliations:** ^1^ Department of Psychology and Behavioral Sciences Zhejiang University Hangzhou 310058 China; ^2^ Department of Clinical Neuroscience Karolinska Institutet Stockholm 17165 Sweden; ^3^ Danish Research Centre for Magnetic Resonance, Centre for Functional and Diagnostic Imaging and Research Copenhagen University Hospital ‐ Amager and Hvidovre Copenhagen 2650 Denmark; ^4^ Centre for Human Brain Health School of Psychology University of Birmingham Birmingham B15 2TT UK; ^5^ Institute for Mental Health School of Psychology University of Birmingham Birmingham B15 2TT UK; ^6^ Social Cognitive and Affective Neuroscience Unit Department of Cognition Emotion and Methods in Psychology Faculty of Psychology University of Vienna Vienna 1010 Austria

**Keywords:** brain‐to‐brain coupling (BtBC), Magnetoencephalography (MEG), observational threat learning, social status, vicarious fear

## Abstract

Survival and adaptation in environments require swift and efficacious learning about what is dangerous. Across species, much of such threat learning is acquired socially, e.g., through the observation of others’ (“demonstrators’”) defensive behaviors. However, the specific neural mechanisms responsible for the integration of information shared between demonstrators and observers remain largely unknown. This dearth of knowledge is addressed by performing magnetoencephalography (MEG) neuroimaging in demonstrator‐observer dyads. A set of stimuli are first shown to a demonstrator whose defensive responses are filmed and later presented to an observer, while neuronal activity is recorded sequentially from both individuals who never interacted directly. These results show that brain‐to‐brain coupling (BtBC) in the fronto‐limbic circuit (including insula, ventromedial, and dorsolateral prefrontal cortex) within demonstrator‐observer dyads predict subsequent expressions of learning in the observer. Importantly, the predictive power of BtBC magnifies when a threat is imminent to the demonstrator. Furthermore, BtBC depends on how observers perceive their social status relative to the demonstrator, likely driven by shared attention and emotion, as bolstered by dyadic pupillary coupling. Taken together, this study describes a brain‐to‐brain mechanism for social threat learning, involving BtBC, which reflects social relationships and predicts adaptive, learned behaviors.

## Graphical abstract

A multi‐brain mechanism for observational threat learning is provided. The study records brain activity in observers learning the threat value of stimuli through watching demonstrators’ aversive reactions to the same stimuli, and finds that demonstrator's and observer's brains couple together in a way that predicts observational threat learning and reflects social hierarchies.

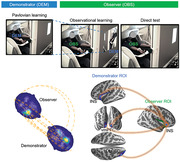

## 1 Introduction

Across many species, successful adaptation to the environment requires learning from conspecifics about what should be avoided and approached.^[^ ^1^, ^2^ ^]^ For example, an observer can quickly learn to associate a neutral stimulus with threat by observing another individual's (a “demonstrator's”) defensive responses to the stimulus.^[^ ^3^, ^4^, ^5^ ^]^ This form of observational or vicarious threat learning has been documented in many non‐human species^[^ ^6^, ^7^, ^8^, ^9^, ^10^ ^]^ and across the human life‐span, from infancy^[^ ^11^, ^12^ ^]^ to adolescence^[^ ^13^, ^14^ ^]^ and adulthood,^[^ ^4^, ^15^, ^16^, ^17^, ^18^ ^]^ serving as an important experimental model to understand the social transfer of value information that can be generalized more broadly.

Several key mechanistic processes subserving such observational threat learning has been captured by research in social, behavioral, and affective neuroscience. For instance, rodents and human studies converge on the conclusion that the amygdala, anterior insula (aINS), and anterior cingulate cortex (ACC) are involved in social threat learning.^[^ ^19^, ^20^, ^21^ ^]^ Most of this research has, however, focused on neural mechanisms in observers, leaving it unexamined how neural systems coordinate across demonstrators and observers engaged in learning.^[^ ^22^ ^]^ Recent efforts leveraging functional near‐infrared spectroscopy (fNIRS) and functional MRI (fMRI) have revealed brain‐to‐brain coupling (BtBC, i.e., the neural systems of two brains that are temporarily coupled^[^ ^23^ ^]^) between individuals during skill^[^ ^24^, ^25^ ^]^ and reward learning.^[^ ^26^ ^]^ But due to limitations in temporal resolution, previous neuroimaging studies have not been able to specify the temporal unfolding of BtBC during observational threat learning. BtBC represents a proxy for correlated neural activity across the brains of two individuals,^[^ ^23^, ^27^, ^28^, ^29^ ^]^ and emerges when, e.g., dyads’ attentions or emotions are shared.^[^ ^30^, ^31^, ^32^ ^]^ Unveiling the temporal dynamics of BtBC will help us understand the real‐time information transfer process during social learning, and eventually aid the development of new strategies to treat learned maladaptive behaviors.

In this study, we aimed at examining the specific multi‐brain mechanisms underlying successful observational threat learning. For this purpose, we used magnetoencephalography (MEG) imaging to record neuronal activity in observers learning the threat value of stimuli through watching demonstrators’ aversive reactions to the same stimuli. Demonstrators’ brain activity was pre‐recorded, allowing for computations of BtBC across demonstrator‐observer dyads. Compared to other approaches (e.g., simultaneous imaging^[^ ^22^, ^33^ ^]^), this MEG‐based sequential imaging approach allowed for a balanced trade‐off between ecological validity and experimental control^[^ ^34^ ^]^ in the study of information transfer in dyads. Importantly, MEG shows superior capability with regard to its spatiotemporal resolution, and enabled us to capture the unfolding of time‐locked neural responses in two individuals during social learning, localized with high spatial resolution to the individual brain anatomy.^[^ ^34^ ^]^

Because the relative status between individuals is important for how they interact and exchange information,^[^ ^35^, ^36^, ^37^ ^]^ we expected relative status to impact the learning process in our paradigm. According to the “low‐status benevolence” hypothesis,^[^ ^38^ ^]^ observers are expected to show stronger empathy (a core constituent of observational threat learning^[^ ^19^, ^39^, ^40^ ^]^) with, pay more attention to, and consequently learn better from, low‐status (vs high‐status) demonstrators. In support of this conjecture, social status has been reported to modulate neural responses of empathy for someone receiving painful stimulation.^[^ ^38^ ^]^ More specifically, activation was observed in regions associated with empathy: the anterior insula and anterior medial cingulate cortex in response to low‐status targets, whereas activation in these regions was attenuated in response to high‐status targets. Moreover, people tend to inhibit emotional sharing or even express schadenfreude toward misfortune high‐status individuals.^[^ ^41^, ^42^ ^]^ Alternatively, according to the “high‐status skilfulness” hypothesis,^[^ ^43^, ^44^, ^45^, ^46^ ^]^ observers should, conversely, pay more attention to, and learn better from, high‐status (vs low‐status) demonstrators, as these demonstrators are assumed to be more skilful and reliable. This hypothesis is supported by research demonstrating that high status have a great social influence and therefore attract more attentional resources compared to those of relatively low status.^[^ ^37^, ^47^ ^]^ Naïve observers might trust a skilled (vs unskilled) demonstrator more to accurately and reliably show adaptive behaviors to threat (e.g., facial expressions and body movements) that reflect the underlying contingency between reaction and consequence, impacting upon the efficiency of observation by learners.^[^ ^43^, ^48^, ^49^ ^]^

The goal of the current study was twofold. First, related to our primary aim, we examined the neural mechanisms (here, BtBC) involved in the transfer of threat information between demonstrators and observers, and how these mechanisms predict successful learning. Given that BtBC can remarkably reflect successful information transfer between individuals,^[^ ^50^, ^51^ ^]^ we expected an enhancement of BtBC during observational acquisition of threat would predict learning outcome (i.e., conditioned responses). In accordance with previous research on observational learning, we expected an enhanced BtBC in brain regions associated with action observation (such as motor areas^[^ ^52^ ^]^), reward processing (such as ventromedial prefrontal cortex, vmPFC,^[^ ^18^ ^]^ anterior insula, aINS,^[^ ^3^ ^]^ and dorsolateral prefrontal cortex, DLPFC^[^ ^16^ ^]^), and social perception and cognition (such as superior temporal sulcus, STS^[^ ^3^, ^5^ ^]^), and primarily engage brain oscillations within low‐frequency bands (such as delta/theta bands, 1 to 8 Hz) that have been implicated in aversive learning.^[^ ^53^, ^54^, ^55^, ^56^, ^57^ ^]^

Second, we tested whether social status would bias observational learning and the associated BtBC. Prior research has shown that a higher level of BtBC is associated with better learning.^[^ ^22^, ^24^, ^58^, ^59^, ^60^, ^61^ ^]^ To the extent that the “low‐status benevolence” hypothesis holds true, we expected stronger learning‐relevant BtBC (expected to characterize threat information transfer from demonstrators to observers) when observers believed they were watching a low‐status demonstrator compared to watching high‐status demonstrator. Alternatively, if the “high‐status skilfulness” hypothesis holds true, we expected stronger learning‐relevant BtBC when observers believed they were watching a high‐status demonstrator compared to watching a low‐status demonstrator.

To these ends, we first verified that the observers had effectively acquired the threat information using physiological measures and computational modeling, and then examined neural responses in the observer using time‐frequency analysis. Importantly, we then focused on BtBC between demonstrators and observers, both at sensor and source levels, and examined its correlation with learning outcomes (i.e., conditioned responses expressed by the observer during the test phase). We used the Circular Correlation Coefficient (CCorr) to compute the BtBC of neural activity, nonnegative matrix factorization (NMF) to cluster BtBC networks, and estimated the cortical source of BtBC through source reconstruction (see Experimental Section). Finally, to advance a better understanding of the functional meaning of BtBC during social learning, a machine learning algorithm was employed to provide a more precise computation for predictive power of BtBC on observational learning outcome across time (as compared to a conventional correlational analysis based on averaged BtBC). Gaze fixation and pupil dilation pattern as major types of adaptive behavior were used to test shared attention and emotion^[^ ^30^, ^31^ ^]^ as sources for BtBC (i.e., whether BtBC was mechanistically driven by shared attentional effort and emotional processing between demonstrator and observer).

## 2 Results

**Figure 1:**
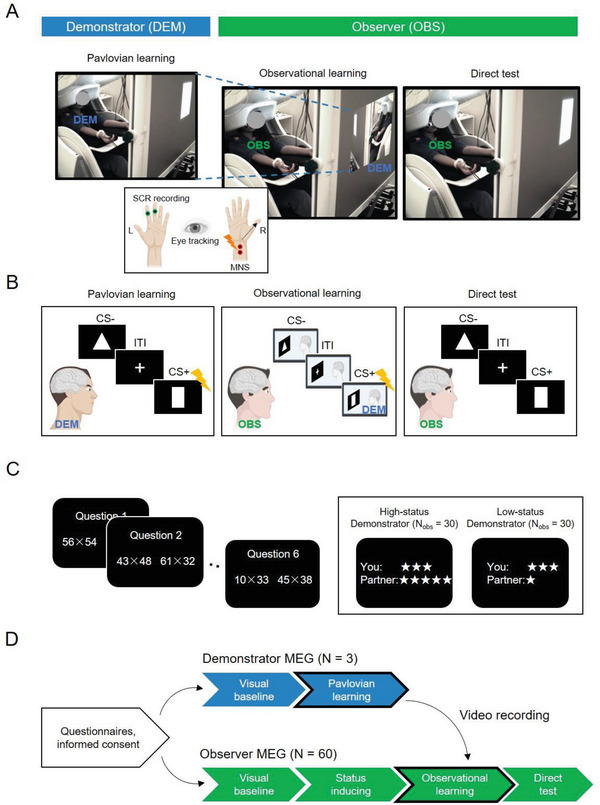
Experimental setup and study protocol. A) The MEG sequential imaging paradigm. The demonstrator (DEM) performed a Pavlovian learning (conditioning) task in an MEG scanner, during which their brain activity was recorded and the whole procedure was filmed. The filmed video was later shown to naïve observers (OBS) in the MEG scanner (observational learning). After observational acquisition, observers performed a direct test in the absence of the demonstrator. Participants’ skin conductance and pupillometry were recorded simultaneously. A median nerve stimulator (MNS) was attached to participant's right wrist for shock delivery (as a threat). Observers never received shocks during observational learning and only received one shock at the final trial of direct test. B) Exemplified stimuli for CS+ and CS‐ during each session. C) Status inducing. Before observational learning, the observer's perceived social status was manipulated via a math competition task. Half of observers believed they were observing a demonstrator with higher/lower status than themselves. D) The overall experimental procedure. Participants also underwent a visual baseline task at the beginning of MEG scanning, during which they watched neutral geometrics without additional information.

To investigate the brain‐to‐brain mechanism for observational learning, we developed a sequential MEG imaging protocol.^[^ ^34^ ^]^ First, we filmed demonstrators and recorded MEG while participants were performing a Pavlovian threat conditioning task (*N* = 3, *Pavlovian learning*), where one conditioned stimulus, CS+, but not the other, CS‐, was probabilistically (62.5%) followed by electrical nerve stimulation (i.e., unconditioned stimulus, US). The demonstrators were naïve participants with naturalistic reactions (i.e., not confederate “actors”). They were not told about the purpose of the study, and this is one of the strengths of our paper, as we were interested in capturing observational learning that occurred naturally between demonstrator and observer. All demonstrators showed successful learning during acquisition in the Pavlovian learning (Figure S1, Supporting Information). Second, we showed these video recordings to naïve observers (N = 60, observational learning) during MEG scanning (**Figure** **1**A,B).

Before observational learning, social status was manipulated via performance payoffs in a math competition task:^[^ ^62^ ^]^ half of the observers were instructed to believe they were observing a higher‐status (HS) demonstrator (*N* = 30), and the other half, a lower‐status (LS) demonstrator (*N* = 30, Figure 1C). The influence of status manipulation on subsequent observational learning could be potentially contaminated by task familiarity or other confounding factors (including learning about physical threats or similar affective processes). Thus, social status was operationalized using a dissimilar and orthogonal task with respect to the main experiment (i.e., math competition vs threat learning). Having a task that is not directly related also provides an even stronger test of the hypothesis that exogenously induced social status would affect learning. Similar to previous experiments,^[^ ^62^, ^63^ ^]^ this manipulation successfully induced feelings of relative social rank (see Experimental Section). After observational learning, observers performed a direct test in the absence of the demonstrator to evaluate their learning outcomes [i.e., CS+/CS‐ differentiation on skin conductance response (SCR) and pupillometry]. Demonstrator and observer visits were on separate days (see Figure 1D for the experimental protocol and Experimental Section for more details). Each demonstrator was paired with ≈20 observers, among which 10 observers were randomly assigned to the LS group and the other 10 observers to the HS group.

### 2.1 Successful Observational Learning in the Sequential MEG Imaging Paradigm

**Figure 2:**
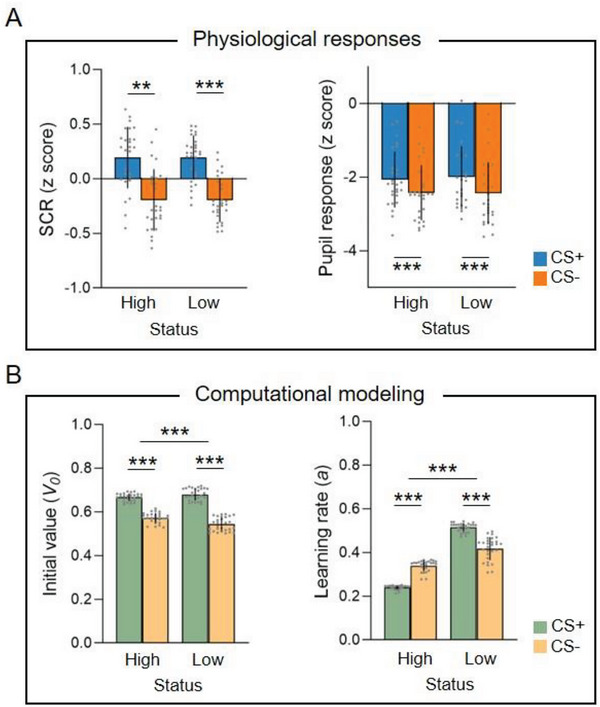
Measures of learning. A) Physiological responses. During direct test, observers showed larger skin conductance response (SCR) and pupil dilation to CS+ versus CS‐, indicating successful learning, irrespective of the demonstrator's social status. The x‐axis represents social status (high vs low); the y‐axis represents z‐scored SCR (left) and pupil dilation (right). B) Computational modeling based on a simple Rescorla‐Wagner reinforcement learning model. Computational modeling of SCR during direct test displayed a larger CS+/CS‐ differentiation on the initial value (*V_0_*) in the low‐status (LS) versus high‐status (HS) groups during Direct Test (left panel). During the Observation phase, the learning rate (α) was enhanced for CS+ compared to CS‐ in the LS group, and this pattern was reversed in the HS group. The x‐axis represents social status (high vs low); the y‐axis represents *V_0_* (left) and α parameters (right) derived from the reinforcement learning model. Error bars denote standard deviations. **P < 0.01, ***P < 0.001.

A linear mixed‐effects model tested and verified successful learning in naïve observers: observers showed significantly higher SCR amplitude for CS+ compared to CS‐ trials (β = 0.39, *SE* = 0.07, *t* = 5.97, *P* < 0.001; **Figure** **2**A, left panel). A similar pattern was observed for CS+/CS‐ differentiation on pupil response (β = 0.45, *SE* = 0.09, *t* = 4.85, *P* < 0.001; Figure 2A, right panel). No significant main effect of social status or interaction were detected (all *t* < 0.66, all *P* > 0.51).

To further uncover the computational basis for observational threat learning, we then used a simple Rescorla‐Wagner reinforcement learning model (winning model after model comparison, see Experimental Section) to quantify the SCR patterns.^[^ ^64^ ^]^ We focused on the model‐derived trial‐by‐trial initial value (*V*_0_), which accounted for initial shock prediction during direct test, and the learning rate (*α*), which elucidated the weight of reward prediction error in value (shock) update during observational learning. Modeling results on initial value revealed main effects of CS type (β = 0.14, *SE* = 0.002, *t* = 73.93, *P* < 0.001) and social status (β = 0.03, *SE* = 0.01, *t* = 4.27, *P* < 0.001), as well as their interaction (β = −0.04, *SE* = 0.003, *t* = −15.76, *P* < 0.001), indicating a significant larger *V_0_* for “CS+_LS_ > CS‐_LS_” compared to “CS+_HS_ > CS‐_HS_” (Figure 2B, left panel). The learning rate during observation learning showed main effects of CS type (β = 0.10, *SE* = 0.005, *t* = 19.76, *P* < 0.001) and social status (β = −0.08, *SE* = 0.01, *t* = −9.34, *P* < 0.001), and importantly, the interaction between them (β = −0.20, *SE* = 0.01, *t* = −27.90, *P* < 0.001). Post‐hoc comparisons indicated that learning rate was significantly higher for CS+ than CS‐ in the LS group, whereas this pattern was reversed in the HS group (Figure 2B, right panel). Taken together, our physiological results demonstrated that naïve observers consistently acquired threat information irrespective of social status. Computational modeling further uncovered that status did, however, bias the initial value during direct test, as well as the learning rate for the extent of value (shock) update during observational learning.

### 2.2 Observational Learning Decreased Widespread Low‐Frequency Neural Response in Observers

**Figure 3:**
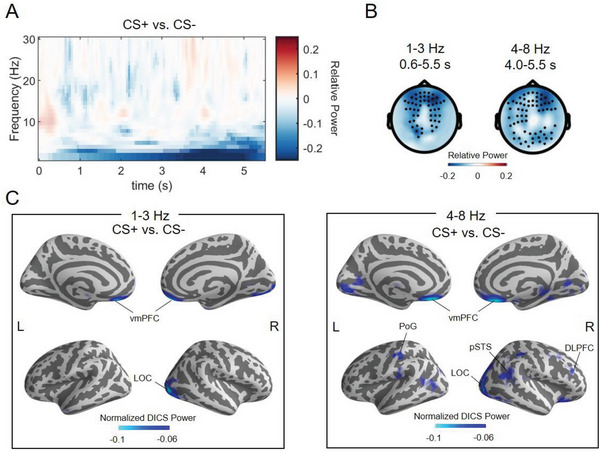
Observers’ brain responses to conditioned stimuli (CS). A) Time‐frequency analyses. Relative power changes in response to CS+ versus CS‐ $\left(\frac{CS+-CS-}{CS-}\right)$ for the observational learning session, averaged over channels that showed significant differences according to cluster‐based permutation test. The color bar denotes relative power changes. B) Sensor‐level topographical plots of the relative power changes in response to CS+ versus CS‐ at delta (1–3 Hz) and theta bands (4–8 Hz), for which cluster‐based permutation test yielded a significant effect. The black dots indicate the channels for which significant effects were found. The color bar denotes relative power changes. C) Source‐level relative power mapping through dynamic imaging of coherent sources (DICS) along the cortex. Contrasts, frequencies, and time windows of interest were matched with those conducted on the sensor level. The color bar represents normalized DICS power [i.e., (Power_CS+_ – Power_CS‐_)/Power_CS‐_]. vmPFC, ventromedial prefrontal cortex; LOC, lateral occipital cortex; DLPFC, dorsolateral prefrontal cortex; PoG, Postcentral gyrus; pSTS, posterior superior temporal sulcus.

To investigate neural activity supporting observational threat learning, we first performed a sensor‐level time‐frequency analysis on observers’ MEG data (a parallel analysis on demonstrators’ brain data can be found in Figure S2 (Supporting Information), albeit no statistical comparisons being applied due to the limited sample size of demonstrators). The non‐parametric cluster‐based permutation tests showed a significant difference between CS+ versus CS‐ (*P* = 0.001), informed by a negative cluster with the frequency ranging from 1–8 Hz and time interval ranging from 0.6–5.5 s post stimulus onset at the fronto‐temporo‐parietal channels (**Figure** **3**A,B). Source‐level power mapping, using dynamic imaging of coherent sources (DICS), indicated that the CS effect (i.e., the 1–8 Hz response from 0.6–5.5 s) involved a decreased power in the ventromedial prefrontal cortex (vmPFC), lateral occipital cortex (LOC), dorsolateral prefrontal cortex (DLPFC), Postcentral gyrus (PoG), and posterior superior temporal sulcus (pSTS) in low‐frequency bands (including delta and theta bands, Figure 3C).

In sum, our results describe a neural signature of observational learning that includes low‐frequency neural oscillations. Similar low‐frequency activity has been demonstrated in aversive learning and empathy.^[^ ^53^, ^54^, ^55^, ^56^, ^57^ ^]^ Time‐frequency analyses did not reveal any significant clusters for the main effect of social status or the interaction (all *P* > 0.13).

### 2.3 Shared Neural Responses Between Demonstrators and Observers

We next examined whether shared neural responses between demonstrators and observers could reflect social status and predict learning outcomes. We used the well‐established Circular Correlation Coefficient (CCorr; see Experimental Section) to measure shared neural responses (i.e., BtBC) between demonstrators and observers. We focused on low frequencies of interest associated with observational threat learning (as detected by the time‐frequency analyses in the previous section) divided into the canonical delta (1–3 Hz) and theta bands (4–8 Hz).

**Figure 4:**
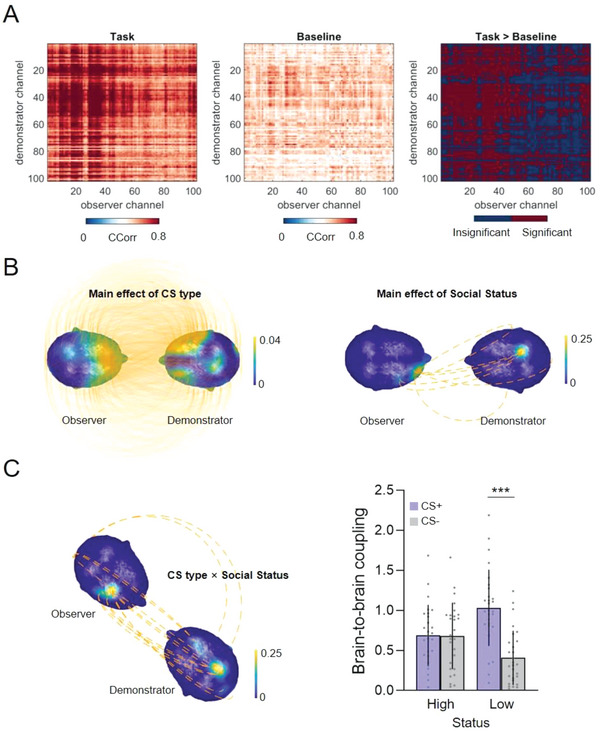
Sensor‐level brain‐to‐brain coupling (BtBC). A) BtBC was computed using Fisher‐z transformed Circular Correlation Coefficient (CCorr). The comparisons between observational learning and baseline helped filtering out channels showing null effects. Only significant channels, for which task induced larger BtBC than baseline, were retained for subsequent analyses. BtBC analyses based on linear mixed‐effects models further revealed a series of main effects of CS type (B, left panel) and social status (B, right panel), and importantly, a CS type × social status interaction (C, left panel). The mean BtBC over channel combinations that showed significant interaction effects was larger for the CS+ relative to CS‐ trials in the low‐status (LS) group, but not in the high‐status (HS) group (C, right panel). The x‐axis denotes social status (high vs low); the y‐axis represents the mean BtBC over channel combinations that showed significant interaction effects (C, right panel). The orange lines over the heads represent statistically significant BtBC between channels in the demonstrator and observer brains (solid line, *P*_FDR_ < 0.05; dashed line, *P*_uncorrected_ < 0.001). The head color indicates the number of BtBC links over a region normalized to the total number of significant BtBC links. Error bars denote standard deviations. ***P < 0.001.

In the first step of the analysis, we compared BtBC during observational learning with BtBC during visual baseline. This analysis filtered out channel combinations (i.e., demonstrator channel paired with observer channel) where BtBC might simply emerge due to common visual inputs and/or environment. We identified 6281 (**Figure** **4**A) and 4330 (Figure S3A, Supporting Information) channel combinations where BtBC during observational learning significantly exceeded that during visual baseline in the delta and theta bands, respectively (all *P*_FDR_ < 0.05).

In the second step, we performed a series of linear mixed‐effects models on channel combinations that survived in the first step. For the delta band, the analysis revealed a series of main effects of CS type (2959 channel combinations, all *P*_FDR_ < 0.05) and social status (8 channel combinations, all *P*_uncorrected_ < 0.001; Figure 4B), and notably, their interaction (13 channel combinations, all *P*_uncorrected_ < 0.001; Figure 4C, left panel). Further analyses on channel combinations which showed interaction effects revealed that CS+_LS_ elicited significantly stronger mean BtBC relative to CS‐_LS_ (CS type × social status: β = −0.61, *SE* = 0.13, *t* = −4.83, *P* < 0.001; Figure 4C, right panel). For the theta band, we found a series of main effects of CS type (630 channel combinations, all *P*_FDR_ < 0.05), indicating that mean BtBC was significantly larger for CS+ than CS‐ trials (β = 0.16, *SE* = 0.02, *t* = 9.85, *P* < 0.001; Figure S3B, Supporting Information). No other effects were observed. As a control, we conducted parallel analyses in the alpha (9–12 Hz) and beta bands (13–30 Hz), neither band of which was among the a‐priori (low‐frequency) bands. No significant results were detected when we compared BtBC across CS type and social status, after multiple comparison corrections in these bands. In the remainder of the paper, we henceforth restricted our analyses to the low‐frequency bands.

In a validation test of BtBC emergence, we found that, overall, BtBC in the low‐frequency bands averaged across all channels from the pseudo‐dataset was significantly weaker than that from our genuine dataset: delta band, pseudo‐dataset versus genuine dataset, 0.28 ± 0.04 versus 0.74 ± 0.18, *t* = 18.85, *P* < 0.001; theta band, 0.15 ± 0.03 versus 0.37 ± 0.12), *t* = 13.26, *P* < 0.001. Furthermore, the results from the pseudo‐dataset did not replicate what we found based on our genuine dataset: No significant main or interaction effects involving social status were detected, after multiple comparison corrections (all *P*_FDR_ > 0.05).

### 2.4 Shared Neural Responses Selectively Predicted Observational Learning

**Figure 5:**
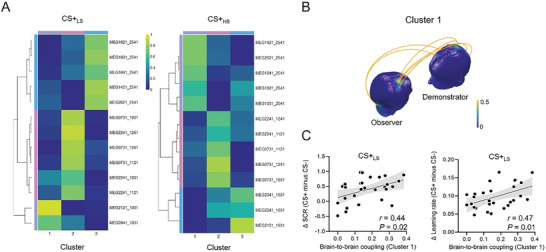
Clustered brain‐to‐brain coupling (BtBC) predicting learning. A) Heatmaps of nonnegative matrix factorization for the CS+ low‐status (CS+_LS_) and CS+ high‐status (CS+_HS_) conditions. The dendrograms in the left part of each heatmap illustrate the organization of the clusters generated by hierarchical clustering. The channel combination names separated by an underscore in the right part of each heatmap denote the demonstrator and observer channels constituting BtBC, respectively. The colors reflect coupling link loadings for each cluster (in arbitrary unit). B) The pattern of BtBC in cluster 1. The head color indicates the number of BtBC links over a region normalized to the total number of significant BtBC links. C) BtBC loadings on cluster 1 predicted differential skin conductance response (SCR) and differential learning rate. Differential responses were defined as the differential response to CS+ versus CS‐.

To further examine the interaction effect in the delta band and to test the relationships of BtBC patterns for each social status condition with learning, we conducted a nonnegative matrix factorization (NMF) analysis on BtBC (**Figure** **5**A). NMF clustered BtBC describing the unique demonstrator‐observer brain network during observational learning.^[^ ^32^ ^]^ Previous research has shown that responses to CS+ during observational learning can successfully predict the expression of learning at a subsequent time, as indicated by converging strands of physiological^[^ ^39^, ^65^, ^66^ ^]^ and neural evidence.^[^ ^67^ ^]^ In line with these findings, we found that responses to the CS+_LS_ condition in NMF‐derived cluster 1 correlated positively with differential SCR (*r* = 0.44, *P* = 0.02) and differential learning rate (*r* = 0.47, *P* = 0.01), indicating that an increase in BtBC as represented by cluster 1 predicted better learning (Figure 5B,C). Clusters 2 and cluster 3 derived from NMF were not correlated with differential SCR (all |*r|* < 0.10, *P* > 0.63) or differential learning rate (all |*r|* < 0.04, *P* > 0.85). In the CS+_HS_ condition, clusters 1–3 were not correlated with differential SCR (all |*r|* < 0.25, *P* > 0.21) or differential learning rate (all |*r|* < 0.18, *P* > 0.36).

### 2.5 Source‐Level Shared Neural Responses Associated with Social Status Predicted Learning

To examine the neural substrates of the brain‐to‐brain mechanism underlying observational threat learning, we next sought to uncover shared neural responses in brain areas associated with learning and social status. We determined our regions of interest (ROI) based on DICS power mapping in this study and previous work.^[^ ^3^ ^]^ Following this, we selected ROIs, including insula (INS), anterior cingulate cortex (ACC), vmPFC, DLPFC, PoG, pSTS, and LOC, in both hemispheres. MEG time series in source space over these ROIs were extracted and submitted to the BtBC analyses.

**Figure 6:**
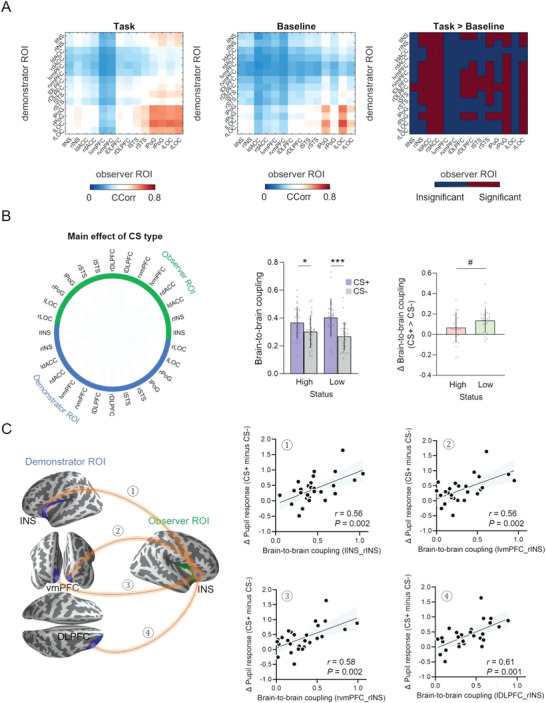
Source‐level brain‐to‐brain coupling (BtBC). A) BtBC was computed using Fisher‐z transformed Circular Correlation Coefficient (CCorr). The comparisons between source‐level BtBC during observational learning (task) and that during visual baseline (baseline). B) Linear mixed‐effects modeling revealed a series of main effects of CS type on BtBC (left panel). The mean BtBC over source regions that showed significant main effects of CS type (y‐axis) was stronger for CS+ than CS‐ trials (middle panel). The low‐status (LS) group exhibited marginally significantly higher differential BtBC (CS+ > CS‐, y‐axis) than the high‐status (HS) group (right panel). C) In the CS+_LS_ condition, demonstrator's left insula (INS, ①), bilateral ventromedial prefrontal cortex (vmPFC, ②③), and right (DLPFC, ④) were consistently coupled to observer's right INS, which further predicted differential pupil response. Error bars denote standard deviations. ^#^ *P* = 0.052, *P < 0.05, ***P < 0.001.

We conducted the BtBC analyses in the delta band. For channel combinations where BtBC during observational learning was significantly stronger than that during visual baseline (**Figure** **6**A), linear mixed‐effects models were conducted for each possible channel combination and revealed a series of main effects of CS type (56 ROI combinations, all *P*_FDR_ < 0.05; Figure 6B, left panel), indicating that there was significantly stronger mean BtBC in CS+ compared to CS‐ trials (β = 0.13, *SE* = 0.02, *t* = 5.46, *P* < 0.001; Figure 6B, middle panel). A planned contrast on mean BtBC revealed a marginally significant difference between “CS+_HS_ > CS‐_HS_” and “CS+_LS_ > CS‐_LS_” (independent *t*‐test, *t* = 1.98, *P* = 0.052; Figure 6B, right panel). No main effect of social status or interactions were detected after FDR correction on either channel combination. Corresponding analyses were conducted also in the theta band (see Figure S4, Supporting Information), with no significant results involving social status or CS type.

We then carried out a series of Pearson correlations to examine in which ROIs BtBC significantly predicted observers’ learning outcome. In the CS+_LS_ condition, we observed that BtBC at lINS_rINS (i.e., demonstrator's left insula and observer's right insula), lvmPFC_rINS, rvmPFC_rINS, and lDLPFC_rINS during observational learning consistently predicted differential pupil responses in observers in the subsequent direct test (all *r* > 0.56, *P*_FDR_ < 0.05; Figure 6C). No significant relationships between BtBC and differential SCR were detected (all *P*_FDR_ > 0.63). In the CS+_HS_ condition, no significant correlations between BtBC and learning were found after FDR correction (all *P*_FDR_ > 0.35). Furthermore, BtBC at lDLPFC_rINS also correlated with observer's self‐reported empathic concern (measured by Interpersonal Reactivity Index^[^ ^68^ ^]^) in the LS group (*r* = 0.41, *P* = 0.03), but not in the HS group (*r* = −0.01, *P* = 0.95).

As a complementary analysis, we additionally tested whether the BtBC effects were driven by one of the demonstrator videos. Specifically, we explicitly tested the effect of demonstrator by including demonstrator ID in the linear mixed‐effects model as an additional fixed factor. We did not find any significant interaction effects involving demonstrator (sensor‐level BtBC: βs < 0.07, *t*s < 0.67, *P*s > 0.50; source‐level BtBC: βs < 0.02, *t*s < 0.64, *P*s > 0.52), indicating that there were no statistical differences in BtBC effects between the three demonstrator videos.

### 2.6 Shared Attention and Emotion as Likely Sources for BtBC Predicting Learning Outcome

**Figure 7:**
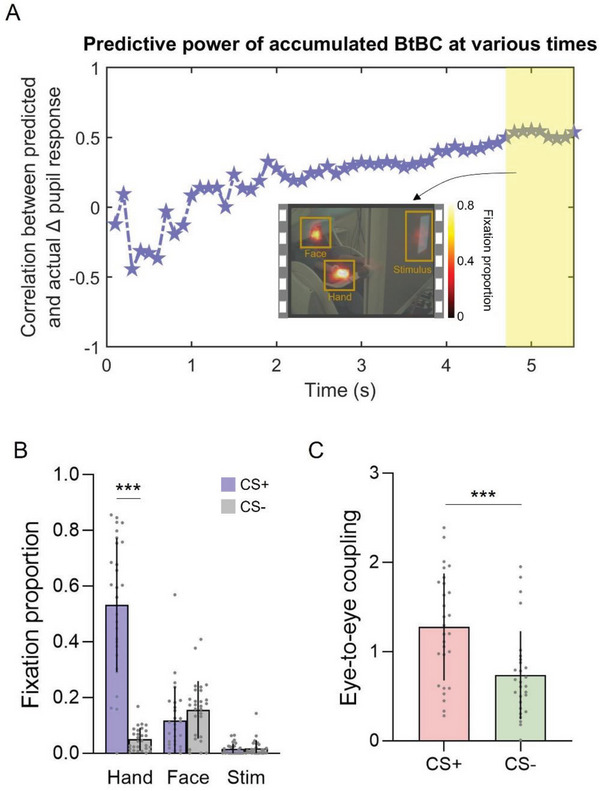
Time‐varying prediction performance. A) Time course of prediction results based on cumulative source‐level BtBC in the CS+ low‐status (CS+_LS_) condition. The yellow shadow marks the time points (4.7–5.5 s) for which cumulative BtBC could significantly predict differential pupil response. Prediction performance was evaluated by the correlation between predicted and actual differential pupil response. An example video frame with fixation proportion in the time window of 4.7–5.5 s is shown. The color bar denotes the fixation proportion (i.e., fixation time at each area of interest normalized to the total fixation time, in arbitrary unit). B) The results of fixation proportion indicate that observers paid more attention to the demonstrator's right hand for the CS+ versus CS‐ trials. The x‐axis denotes areas of interest (hand vs face vs stimulus); the y‐axis represents the fixation proportion. C) Eye‐to‐eye coupling (a corollary of shared attention^[^ ^74^ ^]^ and emotion^[^ ^31^ ^]^) was significantly stronger for CS+ relative to CS‐ trials. The x‐axis denotes CS types; the y‐axis represents eye‐to‐eye coupling computed by Fisher‐z transformed Circular Correlation Coefficient.

Having established that BtBC in the fronto‐limbic circuit can predict learning outcome (as measured by differential pupil response, see the previous section), we further investigate how early that learning outcome at direct test could be decoded from source‐level BtBC at observational learning. To this end, we conducted time‐varying support vector regressions (SVR). Indeed, both empirical^[^ ^69^, ^70^ ^]^ and theoretical^[^ ^71^ ^]^ accounts of threat learning have described its temporal dependency in relationship to predictive events. Therefore, capitalizing on the uniquely high temporal resolution of MEG, rather than averaging BtBC across time, we repeatedly predicted trial‐averaged learning outcome (i.e., pupil response) in the direct phase based on cumulative BtBC in the CS+_LS_ condition for each time point in the observational learning phase. This analysis would inform whether BtBC could predict learning outcome shortly after CS onset or when a threat is imminent (i.e., close to the US). We observed that the prediction performance was improving and reached significance starting at ≈4.7 s after CS onset, immediately preceding the US (note that a US was expected to come 5.5 s post CS onset; all *P*_FDR_ < 0.05, **Figure** **7**A). The prediction accuracy of the model was expressed by the Pearson correlation coefficient between the actual and predicted values.^[^ ^72^, ^73^ ^]^ The mean absolute errors (MAEs) were also reported. Better fit of the prediction model can be characterized by a higher value of correlation coefficient and a lower value of MAE. Our results showed that, the correlation coefficients between predicted and actual values were larger than 0.49, with MAEs smaller than 0.28. We performed a parallel analysis in the CS+_HS_ condition. No significant predictions were found after FDR correction (all *P*_FDR_ > 0.16). These findings indicate that BtBC data are able to predict learning outcome for the LS condition (but not HS) when a threat is imminent to the demonstrator.

To better understand the functional meaning of the BtBC for the prediction of learning outcome in the LS group, we carried out two complementary analyses. First, to ascertain how the observers allocated their attentional focus, we parsed the fixation proportion (i.e., fixation time at each area of interest normalized to the total fixation time) during observational learning. A linear mixed‐effects model demonstrated that the observers pre‐dominantly paid attention to the area including the demonstrator's right hand, which was attached to the stimulator that administered the electric stimulation during CS+ versus CS‐ trials (β = 0.48, *SE* = 0.05, *t* = 10.52, *P* < 0.001; Figure 7B), in the time window of 4.7–5.5 s post CS onset. Other areas of interest did not show this attentional bias (Face: β = −0.04, *SE* = 0.02, *t* = −1.87, *P* = 0.07; Stimulus: β = −0.003, *SE* = 0.006, *t* = −0.43, *P* = 0.67; Figure 7B). In an exploratory analysis, we sought to test whether observer's physiological responses could be predicted by fixation proportion on the demonstrator's face, hand, and the CS stimuli. Results showed that in the LS group, observers’ pupil dilations during CS+ trials were negatively correlated with their fixation proportions on the demonstrator's hand during CS+ trials (*r* = −0.41, uncorrected *P* = 0.03). Second, we computed eye‐to‐eye coupling (a corollary of shared attention^[^ ^74^ ^]^ and emotion^[^ ^31^ ^]^) between demonstrator and observer in the same time window for both CS+ and CS‐ trials. Here, eye‐to‐eye coupling was defined based on the similarity between demonstrator's and observer's pupil dilation that is thought to typically track attentional effort^[^ ^75^ ^]^ and sensitive to emotional peaks.^[^ ^31^ ^]^ Notably, eye‐to‐eye coupling was significantly stronger in the CS+ compared to CS‐ trials (β = 0.54, *SE* = 0.15, *t* = 3.83, *P* < 0.001; Figure 7C). These exploratory analyses indicate a higher level of shared attentional effort and emotional response between observers and demonstrators on CS+ versus CS‐ trials when observers believed they were watching a LS demonstrator.

## 3 Discussion

The present study used a multi‐brain MEG‐imaging approach to understand the neural mechanisms underlying learning through observation, and its dependency on social status. We showed that following the observation of a demonstrator's defensive threat responses, naïve observers successfully acquired, and later expressed, learning (indexed by mean SCR and pupillometry) in the absence of the demonstrator. Although the impact of social status was not visible in these average‐based indices of learning, it was manifested in two important computational properties: initial value and learning rate. On the neural level, we found that CS+ compared to CS‐ conditions downregulated widespread delta and theta oscillatory responses in the observers when watching the demonstrator. Importantly, interbrain analysis identified significantly increased low‐frequency BtBC during observational acquisition of threat compared to the visual baseline for the demonstrator‐observer dyads, suggesting that the alignment of neural processes could not be explained by shared environment or stimulus inputs. Such BtBC in the delta band was greater for CS+ versus CS‐ trials in the LS group but not in the HS group. Strikingly, the social status‐dependent BtBC was also predictive of observers’ ensuing learning outcome (as indicated by SCR and pupillometry) and the predictive power of BtBC was remarkably amplified after ≈4.7 s relative to CS onset (immediately preceding the US). Further analysis on gaze fixation and eye‐to‐eye coupling revealed that BtBC predicting learning outcome was likely to be driven by shared attention and emotion. In the following, we discuss our five key findings.

First, our peripheral physiological results validated successful threat learning (CS+ > CS‐) in naïve observers in a novel sequential MEG imaging paradigm. Compared to conventional video‐based social learning paradigm,^[^ ^5^ ^]^ the use of naïve demonstrators in our paradigm enhanced ecology validity without compromising experimental rigor.^[^ ^34^ ^]^ Moreover, the inclusion of social status, added to the realism by introducing an important social variable present in some forms in most social encounters. Our results showed that social status biased the learning rate during observational acquisition and the initial value in subsequent direct test. These observations suggest that perceived social relationships might affect the fine‐grained dynamic update of threat value predictions as well as acquired prior expectation toward threat. We also observed that the HS and LS groups showed opposite patterns in terms of learning rate, indicating that in the LS group, observers showed faster learning for the CS+ than CS‐ trials, whereas in the HS group, observers showed slower but steadier learning.^[^ ^76^ ^]^ These findings could be interpreted as observers empathize quicker and thus learn from the LS demonstrator and might suggest that observers learn from HS demonstrators through a different strategy, possibly through slowly establishing trust of the demonstrator. In this study, a math competition task was used to manipulate relative social status. While this task has been employed successfully in the past,^[^ ^62^, ^63^, ^77^ ^]^ we cannot definitively determine the experiences of the participants beyond what they reported in their ratings of relative status. It is possible that some participants in fact just recalled the relative number of stars and were unconcerned with status. Yet, this scenario is unlikely based on our manipulation check. Besides, future studies using a more naturalistic procedure (e.g., arm wrestling, tug of war) to induce social status would be a favorable approach to enhance the ecological validity of the observational setting.

Second, observational threat learning downregulated low‐frequency brain oscillatory activity. Specifically, we found decreased oscillatory power for CS+ versus CS‐ trials in the delta (1–3 Hz) and theta (4–8 Hz) frequency bands. We highlight two possible mechanisms through which low‐frequency activity might play a crucial role in observational threat learning. First, the local and distal communications of threat‐related oscillatory activity might shape social learning. This account is supported by previous evidence suggesting that the low‐frequency oscillations represent a mechanism for the coordination of cell assemblies that synchronizes spiking activity and gates interregional information transfer of aversive responses, as demonstrated in both human^[^ ^55^, ^57^ ^]^ and nonhuman animal models.^[^ ^56^ ^]^ Here, we extend the previous proposal by arguing that synchronization of oscillatory activity contributes to information transfer across brains.^[^ ^22^ ^]^ A second possible account for low‐frequency responses to social threat learning would suggest that prefrontal low‐frequency oscillatory power reduction might reflect the modulation of cardiac vagal control that mediates heart rate variability,^[^ ^78^ ^]^ which is known to affect emotional regulation brain networks.^[^ ^79^ ^]^ This perspective is consistent with other accounts suggesting that neural processes associated with emotional processing are involved during observational learning to regulate cognition and adaptive behaviors.^[^ ^80^, ^81^ ^]^ Future research should focus on examining the exact functional meanings of oscillatory power in low‐frequency bands during social threat learning.

Our third main findings was the significantly enhanced low‐frequency BtBC at centro‐frontal channels, which echoed findings in previous studies.^[^ ^82^, ^83^ ^]^ Although there were no direct interactions between the two individuals in the experimental setup, the temporal variation in the observer's brain activity contained information about the corresponding variation in that of the demonstrator, through the video acting as a “medium” supporting transmitted and shared attention and emotion. It could be argued that the relationship between the brain responses of the demonstrator and the observer is merely a correlation resulting from similar independent processes occurring at different time and space in two brains. This viewpoint is however weakened in light of decades of research demonstrating how BtBC reflects unidirectional information transfer from one individual to another during the sequential scanning procedure (Hou et al., 2020; Kostorz et al., 2020; Liu et al., 2017; Nguyen et al., 2021; Stephens et al., 2010). Mitigating this concern further, our validating control analysis confirmed that BtBC was largely attenuated when using biologically‐plausible signals from demonstrator in the BtBC computation. The fact that BtBC was identified for observational learning versus visual baseline allowed us to exclude the possibility that BtBC could simply reflect shared environment or stimulus inputs between demonstrator and observer.^[^ ^22^, ^85^ ^]^ On the source level, observers’ right INS coupled to demonstrators’ left INS, bilateral vmPFC, as well as right DLPFC (all within a fronto‐limbic circuit) in a way that predicted learning outcome. In addition, BtBC involving observers’ rINS correlated with their self‐reported empathic concern, which assesses “other‐oriented” feelings of sympathy and concern for unfortunate others.^[^ ^68^ ^]^ These were only observed in the delta band (but not in any other band) and in the LS (but not HS) group. INS is an important hub of aversive learning network linked to empathic value of pain in observers.^[^ ^3^ ^]^ This involvement bolsters the “low‐status benevolence” hypothesis positing that observers might be more empathic toward low‐status demonstrators. The rationale behind “low‐status benevolence” might be underpinned by the knowledge that others are superior (than oneself) often violates positive self‐concept and aggravates negative feelings due to upward social comparison;^[^ ^41^ ^]^ in contrast, knowing others are inferior is associated with subjective sensitivity to other's pain and affective sharing.^[^ ^38^ ^]^ Affective sharing has been reported as a key aspect supporting observational threat learning, as argued in several previous work.^[^ ^39^, ^40^ ^]^ It is reasonable to infer that the LS (vs HS) group was involved more in the process of affective sharing, leading to stronger learning (as indexed by computational learning rate and initial value). Concerning the functional meaning of vmPFC, previous evidence from learning and decision‐making has shown that vmPFC encodes direct valuation.^[^ ^18^, ^86^ ^]^ A recent patient study also demonstrated a causal role of vmPFC in direct threat conditioning, suggesting that vmPFC is required to generate a sustained conditioned response when anticipating the US during threat learning in humans.^[^ ^87^ ^]^ Our results demonstrate the role of vmPFC in threat learning into a social context. These findings echo previous neuroimaging studies in humans showing shared neuronal patterns of this region in direct and observational learning, suggesting that vmPFC not only represents individuals’ own valuation of social information in learning^[^ ^18^, ^88^ ^]^ but also processes observational outcome prediction errors.^[^ ^16^ ^]^ Like vmPFC, DLPFC activity was commonly involved in direct and observational learning, and typically correlated with expected action prediction errors.^[^ ^16^ ^]^ In support of this, the DLPFC has been implicated in selection of action,^[^ ^89^ ^]^ where DLPFC activity paralleled with uncertainly about which action to select.^[^ ^90^ ^]^ In social situations, DLPFC was thought to be an important driver of emotion regulation.^[^ ^91^ ^]^ A meta‐analysis of fMRI studies of emotional regulation found that increased BOLD signal in DLPFC was associated with strategies that aimed at downregulating negative emotions.^[^ ^91^, ^92^ ^]^ These findings suggest that an integrated neural system that arises from demonstrator and observer, involving the brain's affective, reward, and social hubs, supports observational learning of threat. Future research should aim to corroborate and expand upon our findings by using even more naturalistic and real‐time observational learning paradigms in the MEG (e.g., a demonstrator undergoing Pavlovian learning while an observer watches the entire procedure in the same environment).

Fourth, BtBC was predictive of learning rate and ensuing learning outcomes. In particular, a time‐varying support vector regression model revealed that BtBC predicted learning outcome after several seconds relative to the CS onset (immediately preceding the US). These effects were detectable in the LS group only, suggesting that social status relationship is a key factor shaping the alignment of neural processes across demonstrator and observer. Such neural alignment impacts on the real‐time information transfer and update of threat value, and eventually on the learning outcome.^[^ ^24^ ^]^ The predictive power of BtBC was augmented when a threat was imminent to the demonstrator (close to the timing at which the shock was delivered), suggesting that BtBC supporting social learning of threat value requires a buffering time (rather than immediately after the onset of CSs) to allow the observers integrate endogenous and exogenous cues. The late emergence (related to CS‐onset) of this effect mimics a typical finding in research on Pavlovian fear conditioning where the first 4 s are thought of as an immediate orienting response to a CS, whereas the seconds just before the US onset are considered to depend on more complex cognitive computations, including processing of properties of the CS, US, and their relationship (e.g., expectancy learning and sensory integration).^[^ ^93^, ^94^, ^95^ ^]^ In our paradigm, it is possible that this temporal interval also contains integration of information about the demonstrator, such as their internal affective states (empathy) and relative social status. One alternative explanation for the mechanism of BtBC is that BtBC could reflect the alignment between imagery (simulation) of finger movement in observers and execution of involuntary movement in demonstrators, as these two processes can elicit similar response in the motor system.^[^ ^96^ ^]^ We ruled out this possibility because 1) we did not observe BtBC in the left motor cortices, and 2) we observed that BtBC can predict learning outcomes, reflecting that BtBC is relevant for information transfer beyond sensorimotor alignment.

Finally, our fifth main finding is that the observed BtBC seemed to be driven by shared attention and emotion between the observer and demonstrator. We tested this possibility by parsing observers’ gaze fixation and demonstrator‐observer pupillary coupling. We noticed that when the US was approaching, observers primarily paid attention to demonstrator's right hand where the shock was administered, which is an area that most demonstrators reported that they would pay major attention to during US imminence as well. This suggests that when a threat was imminent to the demonstrator, both demonstrators and observers focused on the upcoming shock delivery, creating an experimental situation favoring the emergence of BtBC. We tested this possibility by parsing pupillary coupling within the dyads. Pupillary dilations reflect the dynamics of conscious attention and emotion; hence spontaneous coupling of pupil dilation patterns has been widely used as a metric of shared processing of attention and emotion.^[^ ^31^ ^]^ Previous studies have shown that collective pupillary coupling reached its crest during the emotional peaks of a narrative,^[^ ^31^ ^]^ and was positively correlated with engagement during conversation.^[^ ^74^ ^]^ Consistent with BtBC patterns, we found CS+ versus CS‐ trials elicited greater eye‐to‐eye coupling in the LS group during observational learning, suggesting shared attention and emotion as likely sources for BtBC.^[^ ^30^ ^]^

In summary, our study investigated the neural mechanism of social threat learning from an interpersonal neuroscience perspective.^[^ ^22^ ^]^ Leveraging a sequential MEG imaging approach, we recorded neuronal activity from demonstrator‐observer dyads during the acquisition of threat value. We found that neuronal activity in the low‐frequency bands coupled across the demonstrator and observer in a way that reflects social status relationships and predicts learning outcomes. Besides providing neuro‐physiological evidence supporting that BtBC is a possible neural marker for social threat learning, our work opens unprecedented opportunities for future research and clinical practices to better understand and treat threat‐ and anxiety‐related disorders as well as maladaptive fears.

## 4 Experimental Section

### Ethics Statement

The study was conducted following the Declaration of Helsinki, with written informed consent obtained from each volunteer before data collection. The study procedure was approved by the Ethics Review Authority in Sweden (2020‐00101). Each participant was reimbursed with 350 SEK (≈$40) for their participation.

### Participants

Sixty adults (37 females, aged 27 ± 5.07) were recruited as observers and three adults (2 females, aged 28 ± 2.89) as demonstrators. Each demonstrator was randomly paired with twenty observers, forming 60 demonstrator‐observer dyads. The choice to limit the number of demonstrators was motivated by our aim to minimize inter‐demonstrator variability and maximize the similarity of the model's style across dyads, which was consistent with previous studies using comparable sequential‐scanning paradigms.^[^ ^25^, ^29^, ^51^, ^72^, ^84^ ^]^ All participants were healthy, right‐handed, and were recruited by advertisements at Karolinska Institutet and surrounding local communities. Data from five observers were dropped out due to insufficient signal quality (*N* = 3) or scanner malfunction during MEG recording (*N* = 2), hence 55 dyads contributed to legit data in the final analyses. No statistical methods were used to pre‐determine the sample size but the sample size was set to match those reported in previous publications.^[^ ^3^, ^97^ ^]^

### Study Procedure

The demonstrators and observers visited the laboratory separately. Demonstrators' visit entailed i) a Pavlovian learning session and ii) a direct test session, whereas observers' visit entailed three sessions: i) social status inducing, ii) observational learning, and iii) direct test (Figure 1). Before the task, participants were attached to SCR and shock electrodes, underwent a personalized shock calibration procedure, and were seated in the MEG scanner (see details in the next sections). The experiment was controlled using Presentation software (NeuroBehavioral Systems, Albany California, USA). At the very beginning of the experiment, both demonstrator and observer were asked to watch two white geometric shapes (a parallelogram and a rhombus) on a black background without any additional information, which served as the visual baseline.

*Demonstrator Sessions*: The formal experiment in demonstrators included two blocks of Pavlovian learning. During the acquisition, two different white geometries served as CSs (a triangle and a rectangle, different from shapes in the visual baseline; Figure 1B). Each CS lasted for 6 s and was presented 16 times, out of which 10 presentations of the CS+ (62.5%) were paired with the US. The US following the CS+ was a 200‐microsiemens (µS) electrical nerve stimulation to the demonstrator (see also the calibration procedure below), which was delivered 5.5 s after the onset of CS presentation. The CS‐ was never co‐terminated with a shock. During the inter‐trial‐interval (ITI), a fixation cross appeared on the center of the screen for 8–12 s. The whole procedure was repeated as a second block, with different CSs (a circle and a pentagon). That is, each block features unique stimulus images, in order to exclude the possibility of carry‐over of learning between blocks. The whole procedure of demonstrator sessions was filmed using an MEG‐compatible video camera. The shooting angle was fixed across participants. Videos recorded during the Pavlovian learning session in demonstrators were extracted using Adobe Premiere Pro CS6. These videos were later shown to naïve observers during the observational learning session.

*Observer Sessions*: The formal experiment in observers was divided into a status‐inducing phase, and two blocks of observational learning and direct test. In the status‐inducing phase, we used a math competition task to manipulate observer's perceived social status (i.e., social rank positions).^[^ ^62^ ^]^ Similar procedures to manipulate social status were initially established by Zink et al.^[^ ^98^ ^]^ and later developed by Hu and colleagues.^[^ ^62^ ^]^ These authors demonstrated that participants strongly engaged in the social status context with this manipulation. It was recognized that social status could have multiple dimensions, including but not limited to socioeconomic status, dominance, prestige, and competence.^[^ ^99^ ^]^ One example of competence‐based social status was the “feelings of rank” in a math competition task, which reflects an individual's level of skill and accomplishment.^[^ ^62^ ^]^ This type of social status manipulation was more easily implemented in a laboratory setting compared to other types. It is important to note, however, that competence‐based social status might not be representative of all forms of social status. Nevertheless, it could be used to simulate scenarios such as exam rankings for admission to different levels of education (related to social learning). Observers were told that more than 50 volunteers had participated in the math competition task, one of which would be randomly selected as the observer's partner (i.e., the demonstrator). Observers were then given 10 s for each of six questions to indicate, by pressing the left or right button on a response box corresponding to the left or right expression, which expression had a greater value (Figure 1C). They were told that performance in this math task would be evaluated by both accuracy and speed, which would be compared with their randomly paired partner. A performance payoff was finally shown based on their estimation performance relative to the demonstrator (in reality the outcome was fixed and manipulated by the experimenter). In the high‐status (HS) group, the demonstrator was associated with five stars, whereas in the low‐status (LS) group, the demonstrator obtained one star only; the observer's ranking was always in the middle with three stars. To clarify, the demonstrators were blind to any ranking information. Half of observers (*N* = 27) believed they were observing a HS demonstrator, and the other half (*N* = 28), a LS demonstrator. As in prior research,^[^ ^62^, ^63^, ^77^ ^]^ each observer was asked to rate the extent to which they perceived their social status as higher or lower than the partner using a 10‐point Likert scale (0 = much lower, 9 = much higher) after completing the entire experiment. The post‐experiment questionnaire revealed that the number of stars used to indicate rank in the math competition strongly influenced participants' ratings of perceived relative social status. An independent *t*‐test confirmed the success of the manipulation in altering feelings of social status (*t* = 12.26, *P* < 0.001). Specifically, observers perceived themselves as higher in status after their partner attained one star (7.11 ± 1.34) as compared to five stars (2.44 ± 1.48).

After that, the observers were told that they would view their partner undergo a learning task and they themselves would go through a similar task thereafter. They watched the video of the demonstrator's responses to the CS‐US pairings to learn which stimulus predicted shock (observational learning session), and then being exposed to the CS in the absence of the demonstrator (direct test session). Note that the observer and demonstrator in a dyad never interacted directly. The observational learning and direct test sessions were repeated as a second block. Videos recorded from each block of the Pavlovian learning sessions in demonstrators were played in the corresponding block of the observational learning sessions in observers (thus, Pavlovian learning in demonstrators and observational learning in observers shared the same experimental timing and trials). At the beginning of the second block, the observers were asked about how many stars they and their partner attained respectively in the math competition task, ensuring that they kept the social rank positions in their minds.

### Electrical Nerve Stimulation

Electrical shocks, comprising of a single 200‐µS pulse, were administrated using an SCG30 stimulator (DeMeTec GmbH, Germany), with felt tips for median nerve stimulation. Before the experiment, participants went through a standard workup procedure to adjust the shock level individually (mean ± SD, 25.32 ± 13.85 mA). This procedure allows each participant to select an intensity that may be perceived as uncomfortable or annoying, but not painful. In the paradigm, shocks will be probabilistically administered (62.5% of CS+ trials) to the right wrist of the demonstrators, when the CS+ appears. Albeit undergoing a similar stimulation setup, the observers won't get any shocks during the experiment (except for the final CS+ trial during the direct test). The shock electrodes were placed over the participant's right wrist and adjusted to a position where receiving a shock could elicit a right thumb movement (Figure 1A). The thumb movement of the demonstrator thus served as a critical cue for observers to acquire the CS‐US contingency.

### Data Acquisitions—MEG Imaging

A Neuromag TRIUX 306‐channel MEG system, including 102 magnetometers and 102 pairs of planar gradiometers, was used for MEG data acquisition. Data were measured at a sampling rate of 1000 Hz, with an online bandpass filter (0.1–330 Hz). The MEG facility was placed inside a two‐layer magnetically shielded room (model Ak3B, Vacuumschmelze GmbH), with internal active shielding to mitigate electromagnetic artifacts. Four head‐position indicator (HPI) coils were attached to the participant's head to monitor head position during MEG recording. The locations for the HPI, anatomical landmarks (e.g., the nasion and the left and right tragus), and additional points (measuring participant's head shape), were determined using a Polhemus Fastrak motion tracker before the measurements, which allowed a co‐registration of the participant's anatomical MRI with the MEG data. Horizontal and vertical electrooculograms (EOG) were recorded with MEG imaging simultaneously.

### Data Acquisitions—Structural MRI

High‐resolution T1‐weighted 3D volume MRI data were acquired using a GE Discovery 3‐T MR scanner (voxel size: 1.0 × 1.0 × 1.0 mm, field of view: 256 mm, repetition time: 2300 ms, echo time: 2.98 ms) from both demonstrators and observers. MRI data were processed with FreeSurfer (version 7) to get cortical reconstruction and volumetric segmentation.^[^ ^100^ ^]^

### Data Acquisitions—Eye Tracking

For eye tracking, an EyeLink 1000 binocular tracker (SR Research, Canada) was used, which measured both eyes binocular at a sampling rate of 500 Hz. Participants were seated at ≈80 cm distance from the eye tracker attached to a projector screen, positioned at ≈100 cm distance. All participants underwent a 5‐point eye‐tracking calibration procedure at the beginning of the experiment.

### Data Acquisitions—Skin Conductance

Participants' electrodermal activity was measured using a BIOPAC MP150 system as the skin conductance signal. Signals were output into the MEG system analog channels and sampled side‐by‐side with the MEG data at a rate of 1000 Hz. SCR electrodes were attached to participants' middle phalanges of the second and third fingers of the left hand (Figure 1A).

### Data Analyses—Learning Assessment

The learning performance was assessed based on conditioned response, which was operationalized as the differential (CS+ minus CS‐) SCR amplitude and/or differential pupil diameter. In addition, a computational modeling approach was applied to provide more mechanistic insights that helped quantifying the underlying processes of observational learning on the trial‐by‐trial basis. For statistical tests, given the fact that each demonstrator formed ≈20 dyads with 20 distinct observers, linear mixed‐effects modeling were used. Data were modelled by CS type (within‐subject predictor: CS+ vs CS‐), social status (between‐subject predictor: high status vs low status), and the interaction of these fixed effects. Random effects were estimated for observer and demonstrator identity, with the former being a nested factor within the latter [*y* ∼ *CS type* * *social status* + (1|*demonstrator*/*observer*)]. Including random factors in the model helped had to account for the nesting of participants in dyads and addressed the concern that effects might be driven by specific demonstrator videos. Linear mixed‐effects modeling was conducted using the *lme4* package in R.^[^ ^101^ ^]^ ANOVAs were conducted to detect the main and interaction effects, using the *anova* function in R. Significance tests on the parameters were performed using Satterthwaite *P* values implemented in the *lmerTest* package.^[^ ^102^, ^103^ ^]^ Post‐hoc contrasts were carried out using the *emmeans* package.^[^ ^104^ ^]^

*SCR*: The raw signals were low‐pass filtered (1 Hz) and high‐pass filtered (0.1 Hz) offline. The SCR was measured for each trial as the largest base‐to‐peak amplitude in µS from 0.5 to 4.5 s following each CS presentation. SCR scores were *z*‐transformed to approach normal distribution.^[^ ^105^ ^]^

*Pupillometry*: Blinks, as revealed by the peak detection on the velocity of the pupil signal and the EyeLink software, were linearly interpolated. Following recent recommendations,^[^ ^106^, ^107^ ^]^ the effects of blinks and saccades on the pupil response were estimated via deconvolution, and omitted these components from the data using linear regression. The pupil time series were then bandpass filtered (0.01–10 Hz), *z*‐transformed, and resampled to 100 Hz. Pupil response for each trial was estimated as the average pupil diameter in the time window of 0–5.5 s relative to stimulus onset, which was baseline corrected by subtracting the mean pupil diameter 0.5 s prior to the onset of the CS presentation.

*Computational Modeling*: The simple Rescorla‐Wagner reinforcement learning model was used for a standard account of error‐driven associative learning.^[^ ^108^ ^]^ *s_t_* was defined as the conditioned stimulus (CS+ or CS‐) on trial *t*, *U_t_* as the US delivered (1 for US, 0 for no US), *V_t_* (*x*) as value (i.e., shock) predictions. The punishment prediction error *δ_t_* was estimated, which measured the difference between the expected and predicted value on trial *t*:

(1) $$V_{t+1}\left(s_{t}\right)=V_{t}\left(s_{t}\right)+\alpha \delta_{t}$$

(2) $$\delta_{t}=U_{t}-V_{t}\left(s_{t}\right)$$

Here, initial values (*V*_0_), was set to 0.5 for the observational learning session, but, importantly, was estimated as a free parameter for the direct test session to capture the carry over learning effect across sessions. For each trial, *s_t_* was updated according to the prediction error. The learning rate α (0 < α < 1) for the value update was a constant free parameter for each participant.

The Rescorla‐Wagner model was fitted to the SCR data.^[^ ^64^ ^]^ The likelihood of SCR on each trial (*SCR_t_*) was modeled as follows:

(3) $$SCR_{t}∼Normal\;\left(\beta_{0}+\beta_{1}V_{t}\left(s_{t}\right),\;\sigma \right)$$

It represents a Gaussian distribution around a mean determined by the scaled value predicted by the model on a given trial *t*, plus a constant term. For completeness, a Rescorla‐Wagner was also tested with *V*_0_ being fixed at 0.5 for the direct test session, and a Pearce‐Hall model^[^ ^54^ ^]^ (both with and without *V*_0_ being fixed for the direct test session). The model described above was consistently the winning model across experimental conditions. Model estimation and model selection were performed using the hierarchical Bayesian approach with the statistical computing language Stan.^[^ ^109^ ^]^

### Data Analyses—Participant's Brain Response to Conditioned Stimuli

The raw MEG data were first submitted to MaxFilter software, which implemented a temporal signal space separation algorithm (buffer length = 10 s, cut‐off correlation = 0.98) to remove external noise from MEG recordings.^[^ ^110^ ^]^ Movement correction was performed by shifting the head position to a position based on the median of the continuous head position during the MEG recording. Further pre‐processing was performed using the Fieldtrip toolbox^[^ ^111^ ^]^ in MATLAB R2019b (MathWorks Inc. Natick, MA). A discrete Fourier transform filter was applied to reduce line noise (for the 50, 100, and 150 Hz). Jump artefacts and artefacts from eye‐blinks were identified and marked in the continuous MEG signals using Fieldtrip's automatic artefact detection algorithms for MEG data and EOG data respectively. Artefacts were removed by zero‐masking the detected data segments (0.11% ± 0.13% of the whole data). An independent component analysis^[^ ^112^ ^]^ was then conducted. Components related to eye‐blinks and heartbeats were identified based on their correlations with EOG or ECG and removed from the data (4.14 ± 8.62 components). The cleaned MEG time series were then chopped into epochs during the time window of −6 to 10 s relative to stimulus onset. Epochs were averaged per session, per condition, for each participant.

Non‐average epochs were submitted to the time‐frequency analysis to estimate participant's response to stimuli. Induced activity was calculated in delta (1–3 Hz), theta (4–8 Hz), alpha (9–12 Hz), and beta (13–30 Hz) frequency bands by time‐frequency decomposition. A complex Morlet wavelet with a Gaussian kernel of 7‐cycle width was used. This procedure was applied to frequencies ranging from 1 to 30 Hz in steps of 1 Hz, in the time window of −2 to 5.5 s relative to stimulus onset. Event‐related power changes were estimated as the percentage change of power relative to the baseline (−2 to −0.2 s relative to stimulus onset). Oscillatory activities within different frequency bands (delta, theta, alpha, and beta) were extracted during the time window of 0–5.5 s post stimulus (i.e., CS) onset. This analysis was done for observers during the observational learning phase. In this study, analyses were focused on MEG signals from magnetometers, which were reported to generate equivalent results as those from gradiometers after signal space separation.^[^ ^113^ ^]^

For statistical analysis of the response to stimuli, non‐parametric cluster‐based permutation tests was done^[^ ^114^ ^]^ on the entire time‐frequency window from 0–5.5 s and 1–30 Hz. First, a dependent‐sample test (a step in the cluster‐based permutation test) on CS+ trials versus CS‐ trials was conducted. Second, to investigate whether oscillatory activities were biased by social status, an independent test between the high‐status (HS) and low‐status (LS) conditions was performed. The interaction between CS type and social status was further tested by conducting a cluster‐based permutation test comparing the differences between the contrast “CS+_HS_ > CS‐_HS_” and the contrast “CS+_LS_ > CS‐_LS_”. Samples were clustered in all tests in connected sets based on temporal, frequency, and channel (*N* > 2) adjacency exceeding alpha level (0.05). Cluster‐level statistics were calculated by taking the sum of the *t*‐values within the cluster with was calculated across randomly shuffling of condition labels. The permutations were performed 1000 times. Cluster statistics exceeding 0.95 percentile (two‐tailed) of the permutation distribution are considered significant (alpha = 0.05).

Dynamic imaging of coherent sources (DICS) was calculated to map out spectral power along the cortex and to find sources of the significant time‐frequency responses. Time and frequency windows were matched with those on the sensor level. The DICS cortical power map was computed as the percentage change of power [i.e., (Power_CS+_ – Power_CS‐_)/Power_CS‐_]. Each individual brain was morphed to an average brain in FreeSurfer to get the group‐level results.

### Data Analyses—Brain‐to‐Brain Coupling (BtBC)

Brain‐to‐brain coupling (BtBC) was estimated using Circular Correlation Coefficient (CCorr).^[^ ^115^ ^]^ CCorr is a measure of the circular covariance of differences between the observed phase and the mean phase.^[^ ^116^ ^]^ It is defined as follows:

(4) $$CCorr_{x,y}=\frac{\sum_{t\;=\;1}^{N}\sin\left(x-\bar{x}\right)\sin\left(y-\bar{y}\right)}{\sqrt{\sum_{t\;=\;1}^{N}sin^{2}\left(x-\bar{x}\right)sin^{2}\left(y-\bar{y}\right)}}$$

In the formula, $\bar{x}$ and $\bar{y}$ are the mean phases for channels from the demonstrator and the observer in a pair, respectively. According to simulations, CCorr was much more robust to coincidental coupling, compared to, e.g., phase‐locking value, when analyzing BtBC data.^[^ ^116^ ^]^ CCorr was computed for the time window of 0–5.5 s post stimulus onset (during observational learning for observers, and during Pavlovian learning for demonstrators), and for each of frequencies that showed significant effect in time‐frequency analysis. Phases for each specific narrow frequency bands were estimated using Hilbert transformation. CCorr values were then normalized by Fisher's *z* transformation and converted into absolute values.^[^ ^32^, ^117^ ^]^ CCorr values ranged from 0 (totally decoupled) to 1 (perfectly coupled), and was calculated using the CircStat toolbox for MATLAB.^[^ ^118^ ^]^

For statistical analyses, linear mixed‐effects modeling was performed to account for the nested data structure for each combination of demonstrator‐observer channels (102 × 102 = 10 404 channel combinations in total). To reduce the number of statistical tests, the following two steps were used. First, the task BtBC (BtBC during observational learning) was compared with baseline BtBC (BtBC for visual baseline, during which participants were watching geometries without additional information) for each channel combination. Channel combinations were focused for which task BtBC was significantly larger than baseline BtBC. This step, following recent recommendations,^[^ ^32^, ^119^ ^]^ allowed to filter out channel combinations with null effect and mitigate the concern that observed BtBC effects were simply due to similar environment or inputs. Only significant channel combinations survived from the first step were analyzed in the second step, in which BtBC was statistically compared across CS types and social status. FDR correction (*P*_FDR_ < 0.05) was applied to control for the multiple testing.^[^ ^120^ ^]^ Channels exhibiting a significant effect without correction (*P*_uncorrected_ < 0.001) were also reported. For visualization of BtBC that showed main or interaction effects, a custom script (dualheadnetplot.m) was used in MATLAB.^[^ ^121^ ^]^

To validate this approach, a pseudo‐dataset of the demonstrator's brain signals was generated by interpolating random time points from multiple demonstrators, which aligns with biological‐plausible principles. For example, the first data point was taken from demonstrator 1, followed by the second data point from demonstrator 3, the third data point from demonstrator 1, the fourth data point from demonstrator 2, and so on in a serially ordered manner. BtBC was then re‐estimated using the pseudo‐sequence.

### Data Analyses—Clustered BtBC

In the next step, BtBC networks (associated with significant interaction effects from BtBC statistical analyses above) during CS+_HS_ and CS+_LS_ were respectively clustered using nonnegative matrix factorization (NMF).^[^ ^122^ ^]^ To reach stable results, NMF was ran 1000 times. Factorization rank (i.e., the number of the cluster, *N* = 3) was determined by taking the first value of rank for which the cophenetic coefficient starts decreasing, according to the “Brunet” version of NMF.^[^ ^123^ ^]^ The relationship between clustered BtBC and learning was tested using Pearson correlation analyses.

### Data Analyses—Source‐Level BtBC

To find the cortical source of BtBC, source reconstruction was then applied to the data. To this end, noise weighted minimum‐norm estimates (MNE) were used for calculating BtBC in source space. The cortically constrained dynamic statistical parametric mapping (dSPM)^[^ ^124^ ^]^ of the MEG data was estimated. The pre‐processed data were imported into MNE‐Python (version 0.23.4).^[^ ^125^ ^]^ Participant's structural MRI was used to generate the head model and co‐registered with the MEG data. The forward solution was estimated from a source space of 5124 evenly spaced points and a boundary‐element model (BEM) volume conductor model derived from the subject's anatomical MRI. The inverse solution was calculated based on the forward solution, with the noise covariance matrix estimated from 2 min of empty room data recorded before the experiment.

MEG time series was derived in source space from the dSPM source reconstructions, which were then exported to MATLAB for BtBC analysis. Regions of interest were defined based on anatomical labels in the Desikan‐Killiany Atlas.^[^ ^126^ ^]^ The ROI selections aimed to match the regions showing pronounced neural activity in DICS power mapping. Five regions in both hemispheres were initially included: vmPFC (atlas label: “medial orbitofrontal”), DLPFC (“rostral middle frontal”), pSTS (“superior temporal”), PoG (“postcentral”), and LOC (“lateral occipital”). Furthermore, considering the critical roles of INS (“insula”) and ACC (“caudal anterior cingulate”) in social threat learning,^[^ ^3^ ^]^ we additionally included both.

To probe the relationship between source‐level BtBC and learning, a series of Pearson correlations on each ROI was conducted. For each group, FDR correction (*P*_FDR_ < 0.05) was applied to control for multiple comparisons.^[^ ^120^ ^]^

### Data Analyses—Predicting Learning from BtBC

The time course of BtBC for CS+ was averaged over the source‐level ROIs that showed significant correlations with learning outcome. The cumulative BtBC, which showed more stable prediction accuracy compared to moment‐to‐moment BtBC data,^[^ ^127^, ^128^ ^]^ across the time was used as the predictor. Specifically, learning was repeatedly predicted in the direct test based on time‐cumulative BtBC in the observational learning from 0.1 to 5.5 s (post stimulus onset) with a time increment of 0.1 s (the parameter of time increment has been tested in the previous work^[^ ^128^ ^]^). The cumulative BtBC at time point *k* was computed as a sum of BtBC at time points from 1 to *k*:

(5) $$BtBC_{cum}=\sum_{i\;=\;1}^{i\;=\;k}BtBC_{i}$$

The support vector regression (SVR) was used to predict learning outcome at each time point. The training dataset was trained by *ε*‐SVR with the radial basis function (RBF). A leave‐one‐dyad‐out cross‐validation was used. Prediction performance of time‐varying SVR was estimated by the Pearson correlation coefficient between predicted and actual learning outcome,^[^ ^72^, ^73^ ^]^ with a series of *P*‐values FDR corrected.^[^ ^120^ ^]^ Mean absolute errors (MAE) were also reported for evaluation of prediction performance. SVR were carried out using the *libsvm* toolbox (version 2.3) in MATLAB.

To provide a better understanding of cumulative BtBC predicting learning, behavioral significance was also parsed at time points that showed satisfactory prediction performance (*P*_FDR_ < 0.05). Two complementary analyses were conducted. First, it was aimed at determining which part(s) of the video the observers were focusing on. Fixation behaviors were identified with the EyeLink software, and analyzed using custom scripts in MATLAB. Three rectangular areas of interest (AOI) were defined (Figure 6): i) the CS on the projector screen, ii) the demonstrator's face, and iii) the demonstrator's right hand entailing the electrical nerve stimulation. Fixation time at each AOI was normalized to the total fixation time at the screen. Second, it was evaluated whether the observers built shared attention^[^ ^31^, ^74^ ^]^ and emotion^[^ ^31^ ^]^ with the demonstrators. To this end, eye‐to‐eye coupling was estimated by computing the CCorr (same as that for BtBC computations) between time series of pupil response between demonstrators and observers.

## Conflict of Interest

The authors declare no conflict of interest.

## Author Contributions

Y.P. and A.O. conceived the initial research idea. D.L. and A.O. supervised the project. Y.P. acquired the data. Y.P., M.C.V., and D.L. implemented the MEG setup. Y.P. performed data analyses, with inputs from M.C.V. on MEG data analyses and L.Z. on computational modeling. Y.P., M.C.V., L.Z., D.L., and A.O. interpreted the results and wrote the manuscript.

## Supporting information

Supporting Information

## Data Availability

The data that support the findings of this study are available from the corresponding author upon reasonable request.
