# Supplementary material for: A Brain‐To‐Brain Mechanism for Social Transmission of Threat Learning
Source: Adv Sci (Weinh). 2023 Aug 6;10(28):2304037. doi: 10.1002/advs.202304037 (PMC10558655; doi:10.1002/advs.202304037)
Supplement: Supplementary file 1 — Supporting Information [file ADVS-10-2304037-s001.pdf]

ADVANCED SCIENCE
Open Access

Supporting Information

for *Adv. Sci.*, DOI 10.1002/advs.202304037

A Brain-To-Brain Mechanism for Social Transmission of Threat Learning

Yafeng Pan, Mikkel C. Vinding, Lei Zhang, Daniel Lundqvist and Andreas Olsson**

Supporting information

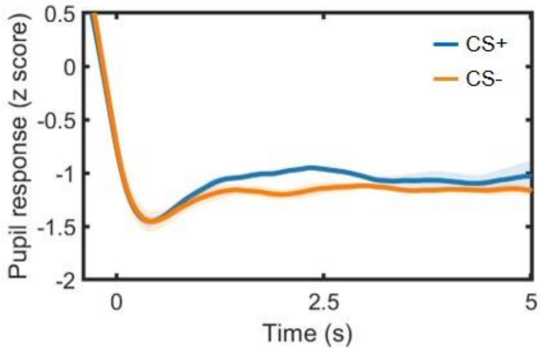

Figure S1. Successful learning in demonstrators. During acquisition, demonstrators (N = 3) showed larger pupil dilation to CS+ vs. CS-, indicating successful learning. 0 s corresponds to the CS onset. Shadows denote standard errors of the mean.

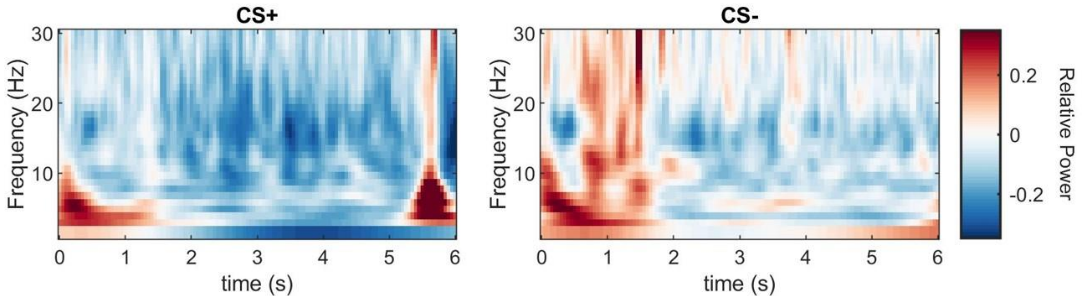

Figure S2. Single-brain responses in demonstrators (N = 3). Relative power changes (task minus baseline) in response to CS+ and CS- for the observational learning session, averaged over all channels. 0 s corresponds to the CS onset, whereas 5.5 s represents the US onset.

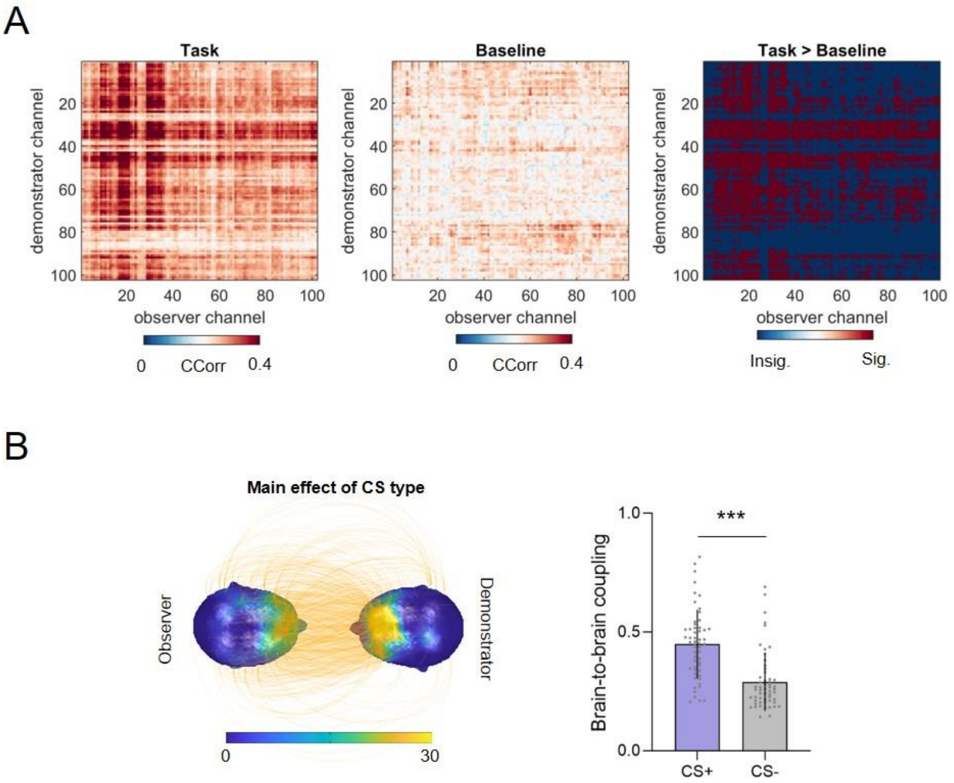

Figure S3. Sensor-level brain-to-brain coupling (BtBC) in the theta band (4-8 Hz). (A) The comparisons between task (observational learning) and baseline (visual baseline) helped filtering out channels showing null effects. Only significant channels, for which task induced larger BtBC than baseline, were retained for subsequent analyses. BtBC analyses further revealed a series of main effects of CS type (**B**, left panel). The mean BtBC over channel combinations that showed main effects of CS type was larger for the CS+ relative to CS- trials (B, right panel). The orange lines over the heads represent statistically significant BtBC between channels in the demonstrator and observer brains (P_{FDR} < 0.05). The head color indicates the number of BtBC links over a region normalized to the total number of significant BtBC. Error bars denote standard deviations. ***P < 0.001.

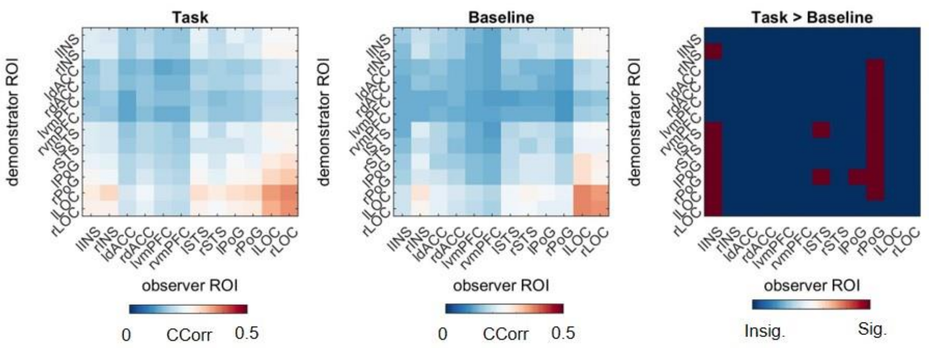

Figure S4. Source-level brain-to-brain coupling (BtBC) in the theta band (4-8 Hz). The comparisons between source-level BtBC during observational learning (task) and that during visual baseline (baseline). Though these comparisons detected stronger BtBC during observational learning (task) compared to that during visual baseline, subsequent analyses did not reveal any significant results involving social status or CS type.
